# Gut Microbiome Influences the Efficacy of PD-1 Antibody Immunotherapy on MSS-Type Colorectal Cancer via Metabolic Pathway

**DOI:** 10.3389/fmicb.2020.00814

**Published:** 2020-04-30

**Authors:** Xinjian Xu, Ji Lv, Fang Guo, Jing Li, Yitao Jia, Da Jiang, Na Wang, Chao Zhang, Lingyu Kong, Yabin Liu, Yanni Zhang, Jian Lv, Zhongxin Li

**Affiliations:** ^1^Second Department of Surgery, The Fourth Hospital of Hebei Medical University, Shijiazhuang, China; ^2^Department of Surgery, First Hospital of Qinhuangdao, Qinhuangdao, China; ^3^Department of Pharmacology, Hebei Medical University, Shijiazhuang, China; ^4^Department of Traditional Chinese Medicine, The Fourth Hospital of Hebei Medical University, Shijiazhuang, China; ^5^College of Combine Traditional Chinese and Western Medicine, Hebei Medical University, Shijiazhuang, China; ^6^Third Department of Oncology, Hebei General Hospital, Shijiazhuang, China; ^7^Department of Oncology, The Fourth Hospital of Hebei Medical University, Shijiazhuang, China; ^8^Department of Clinical Laboratory, The Fourth Hospital of Hebei Medical University, Shijiazhuang, China

**Keywords:** gut microbiota, metabolic pathway, PD-1 antibody, immunotherapy, MSS-type CRC

## Abstract

Colorectal cancer (CRC) appears to be rather refractory to checkpoint blockers except the patient with deficient in mismatch repair (dMMR). Therefore, new advances in the treatment of most mismatch repair proficiency (pMMR) (also known as microsatellite stability, MSS) type of CRC patients are considered to be an important clinical issue associated with programmed death 1 (PD-1) inhibitors. In the present study, we evaluated the effects of gut microbiome of MSS-type CRC tumor-bearing mice treated with different antibiotics on PD-1 antibody immunotherapy response. Our results confirmed that the gut microbiome played a key role in the treatment of CT26 tumor-bearing mice with PD-1 antibody. After PD-1 antibody treatment, the injection of antibiotics counteracted the efficacy of PD-1 antibody in inhibiting tumor growth when compared with the Control group (mice were treated with sterile drinking water). Bacteroides_sp._CAG:927 and Bacteroidales_S24-7 were enriched in Control group. Bacteroides_sp._CAG:927, Prevotella_sp._CAG: 1031 and Bacteroides were enriched in Coli group [mice were treated with colistin (2 mg/ml)], Prevotella_sp._CAG:485 and Akkermansia_muciniphila were enriched in Vanc group [mice were treated with vancomycin alone (0.25 mg/ml)]. The metabolites were enriched in the glycerophospholipid metabolic pathway consistent with the metagenomic prediction pathway in Vanc group, Prevotella_sp._CAG:485 and Akkermansia may maintain the normal efficacy of PD-1 antibody by affecting the metabolism of glycerophospholipid. Changes in gut microbiome leaded to changes in glycerophospholipid metabolism level, which may affect the expression of immune-related cytokines IFN-γ and IL-2 in the tumor microenvironment, resulting in a different therapeutic effect of PD-1 antibody. Our findings show that changes in the gut microbiome affect the glycerophospholipid metabolic pathway, thereby regulating the therapeutic potential of PD-1 antibody in the immunotherapy of MSS-type CRC tumor-bearing mice.

## Introduction

Despite significant advances in surgical, chemotherapy and biological therapy, human CRC remains the fourth leading cause of cancer-related death in the world (Ferlay et al., 2015). From tumorigenesis to treatment, the immune system plays a complex and multifaceted role in cancer, affecting all aspects of cancer. CRC is under robust immunosurveillance, and one of the hallmarks of CRC is its ability to evade the activity of the immune system (Markman and Shiao, 2015). CRC can evade the immune system in several ways (Hanahan and Weinberg, 2011). PD-1 protein mainly inhibits effector T-cell activity within tissue and tumors (Dong et al., 2002), and one of its ligands, PD-L1, has a very broad range of expression in DC, macrophages, T-cells, B cells, epithelial and endothelial cells (Eppihimer et al., 2002). PD-1/PD-L1 play a vital role in the ability of tumor cells to evade the host’s immune system. Recently, blocking the immunological checkpoints mediated by PD-1 or its ligands have led to significant clinical responses in patients with different types of cancers, such as melanoma (Ribas et al., 2015) and non-small cell lung cancer (Brahmer et al., 2015). Nonetheless, the role of immunotherapy in the treatment of unresectable or metastatic CRC is currently limited (Brahmer et al., 2012). Recent clinical evidence has demonstrated that only a minority of patients with CRC demonstrated significant responses to PD-1 blockade (Lynch and Murphy, 2016), and these solitary patient with CRC who had responded to the PD-1 inhibitor were dMMR (also known as MSI) (Aguiar et al., 2015). However, the treatment of most pMMR CRC patients is a major challenge for CRC immunotherapy (Dudley et al., 2016). Studies have shown that immune CPI monotherapy, including PD-1 blockade or anti-CTLA-4 therapy, is associated with virtually no activity in patients with pMMR/non-MSI-H metastatic CRC (Asaoka et al., 2015; Le et al., 2017), while approximately 10–15% of CRC tumors are associated with MSI (Boland and Goel, 2010).

Therefore, new advances in MSS type CRC therapy are considered to be an important clinical issue associated with PD-1 inhibitors (Boland and Ma, 2017). As is known to all, there is a wide range of microbial communities in the human gut, and the composition of intestinal bacterial flora plays key roles in the regulation of the immune system and inter-patient heterogeneity in tumor immunity (Miyauchi et al., 2017). Recently, more and more research showed that anti-tumor immune responses may be influenced by gut microbiome through innate and adaptive immunity, and the anti-tumor immune response can be improved by regulating the intestinal microbiota (Paulos et al., 2007; Sivan et al., 2015). However, the way in which the gut microbiome affects the therapeutic effect of PD-1 antibody on MSS-type CRC needs further exploration.

Studies on the composition and function of gut microbiome have revealed a lot about the role of intestinal flora in human health, but only a few flora metabolites that affect host physiology have been identified. These flora metabolites include short-chain fatty acids produced by the degradation of dietary fiber by the flora, secondary bile acids produced by bacteria in the colon to convert primary bile acids, phosphatidylcholine/choline/L-carnitine and other nutrients that are co-metabolized by the flora-host TMAO (Collins and Patterson, 2020). These studies have elaborated from the molecular level that the intestinal single species-Clostridium sporogenes can change the content of certain chemical substances in mouse blood, and then affect the related functions of the intestinal tract and immune system (Dodd et al., 2017; Lee et al., 2019).

Previous studies have shown that CT26 cells belong to the MSS-type mouse colorectal tumor cell line and are non-sensitive to immune checkpoint blocking (Castle et al., 2014; Kim et al., 2014). In this study, we used mice treated with different antibiotics to establish CT26 CRC xenograft model to compare the relative efficacy of different antibiotic groups after PD-1 antibody immunotherapy. The aim was to investigate the effect of gut microbiome on the immunological efficacy of PD-1 antibody in the treatment of MSS-type CRC through metabolic pathway.

## Materials and Methods

### Establishment of CT26 Xenograft Model in Different Antibiotic Backgrounds

Male BALB/c mice (Purchased from The Fourth Hospital of Hebei Medical University), SPF and inbreeding, aged from 7 weeks (Vetizou et al., 2015; Zhao et al., 2017), each of the 8 mice was randomly divided into one cage. Mice were treated with antibiotics 2–3 weeks prior to tumor implantation and continued until the end of the experiment. The mice were divided into four following 4 groups. Group 1 mice were treated with a mix of ampicillin (1 mg/ml), streptomycin (5 mg/ml), and colistin (1 mg/ml) in sterile drinking water (Asc group) (Al-Qadsy et al., 2020; Yu and Zhao, 2020). Group 2 mice were treated with vancomycin alone (0.25 mg/ml) in sterile drinking water (Vanc group) (Liu et al., 2020). Group 3 mice were treated with colistin (2 mg/ml) in sterile drinking water (Coli group) (Lee et al., 2019). Group 4 mice were treated only with sterile drinking water (Control group). The MSS-type mouse colon carcinoma cell lines colon 26 (CT26) (purchased from Procell Life Science & Technology Co., Ltd. Cat:CL-0071) were cultured in RPMI1640 medium (GIBCO) supplemented with 10% heat-inactivated fetal calf serum at 37°C in a 5% CO_2_ incubator. According to the above group, mice were subcutaneously injected into the right axillary with 0.25 × 10^7^ live CT26 tumor cells. To observe the tumor growth in different groups, and the tumor volume was measured with caliper. When tumors reached a size of 50mm^3^ (9 weeks old), mice were injected intraperitoneally (i.p) with 250 μg of anti-mouse PD-1 antibody (CD279) (BioCell, Cat#:BE0273) or isotype control mAb (anti-rat IgG2a, Clone RG7/1.30). Mice were injected 5 times at 3-day intervals with anti-mouse PD-1 antibody (CD279) or isotype control, and tumor size was routinely monitored by means of a caliper. At the beginning and end of the experiment, fecal samples and blood samples (Supplementary Table S1) were collected before and after injection of PD-1 antibody for subsequent 16S sequencing/metagenomic WGS sequencing and metabolomics profiling, respectively.

### 16S rRNA Sequencing and Analysis

16S rRNA gene sequencing was performed on mouse fecal pellets before (9 weeks) and after injection of PD-1 antibody (9 weeks + 21 days). Using PowerSoil DNA Isolation Kit (MO BIO Laboratories, Carlsbad, CA, United States) according to the manufacturer’s instructions, to isolate DNA from mouse fecal pellets. Purified DNA (1 ng) was used to amplify the 16S rDNA V4 region using the 515F and 806R primers on ABI GeneAmp^®^ 9700 platform (Caporaso et al., 2012), followed by sequenced on the MiSeq platform (Illumina, Inc., San Diego, CA, United States) to generate 2 × 250 bp paired-end reads.

The raw data were merged and quality filtered using FLASH (Fast Length Adjustment of Short reads, v1.2.11). The data were further analyzed using Quantitative Insights into Microbial Ecology (QIIME) software package. Usearch (vsesion 7.0) was used to obtain the operational taxonomic unit (OTU) with 97% sequence similarity and RDP classifier (version 2.2) was used to classify OTUs at a given taxonomic rank.

### Metagenomic Whole Genome Shotgun (WGS) Sequencing and Analysis

Extracted bacterial genomic DNA for 16S rRNA gene component analysis for WGS sequencing. Briefly, 1 μg DNA from mouse fecal pellets was fragmented using the Covaris M220 sonication device, then using the AxyPrep Mag PCR clean up Kit according to the manufacturer’s instructions to construct the sequencing library. The resulting DNA was pooled, quantified and sequenced using an Hiseq2500 platform (Illumina, Inc., San Diego, CA, United States) to generate 2 × 126 bp paired-end reads.

The length of each read has been trimmed with Sickle. Read sequences aligned with the mouse genome were also deleted. Then this set of high-quality reads was used for further analysis. The Illumina short reads were assembled into contigs using IDBA-UD/Megahit (Peng et al., 2012). Genes were predicted on the contigs with MetaGene (Zhu et al., 2010) and the NR gene catalog was constructed with CD-HIT (Li and Godzik, 2006). In order to assess the relative gene abundance, reads were compared to the SOAPaligner gene catalog. Functional annotation was further performed. The putative amino acid of gene catalog was aligned against the proteins/domains in COG and KEGG databases using blastp.

### Non-targeted Metabolomic and Lipidomic Analyses by Ultra-High Performance Liquid Chromatography-Mass Spectrometer (UPLC-MS)

Plasma samples (100 μL) were precipitated by addition of 3 volumes of organic solvent [acetonitrile (1:3 v:v)] pre-cooled to −20°C. After vortex mixed for 1 min, overnight at −20°C and then centrifuged at 14,000 *g* for 20 min. Collected the supernatant and diluted to 50% and UPLC-MS non-targeted metabolomic analyzed. Organic phases were collected and reconstituted in isopropanol/acetonitrile/H_2_O (1:1:1 v:v:v) and UPLC-MS non-targeted lipidomic analyzed.

Precipitated samples (non-targeted metabolomic analyzed) were injected onto a Waters HSS T3 column using a Waters Acquity^TM^ UPLC system equipped with a Waters Xevo^TM^ G2-XS Qtof. Flow rate was 450 μL/min. The mobile phase A consists of 0.1% formic acid in water and mobile phase B consists of 0.1% formic acid in acetonitrile. After separation by UPLC, mass spectrometry was performed using Waters Xevo^TM^ G2-XS Qtof. In positive ion-mode, the mass spectrometry of the optimal conditions was as follows:cone voltage at 24 V, capillary voltage 2.5 kV, source temperature was 100°C, cone gas flow was 50 L/h and desolvation gas flow was 800 L/h. Acquisition time was performed from m/z 50 to 1,500 Da. In negative ion mode, the mass spectrometry parameters were: cone voltage at 25 V, capillary voltage 2.5 kV, source temperature was 100°C, cone gas flow at 10 L/h and desolvation gas flow at 600 L/h. Acquisition time was performed from m/z 50 to 1,500 Da.

The extracted samples (organic phase) (non-targeted lipidomic analyzed) were injected onto a Waters CSH C18 column using a Waters Acquity^TM^ UPLC system equipped with a Waters Xevo^TM^ G2-XS Qtof. The flow rate was 400 μL/min. The mobile phase A consists of acetonitrile/H_2_O (60:40, v:v) mixed with 10 mM ammonium formate and 0.1% formic acid and mobile phase B consists of isopropanol/acetonitrile (90:10, v:v) mixed with 10 mM ammonium formate and 0.1% formic acid. This chromatographic approach allowed an effective separation of the different lipid species. Mass spectrometry was further performed using Waters Xevo^TM^ G2-XS Qtof. In positive ion-mode, the mass spectrometry of the optimal conditions was as follows: cone voltage at 25 V, capillary voltage 2.5 kV, source temperature was 100°C, cone gas flow was 10 L/h and desolvation gas flow was 600 L/h. Acquisition time was performed from m/z 100 to 2,000 Da. In negative ion mode, the mass spectrometry parameters were: cone voltage at 40 V, capillary voltage 2 kV, source temperature was 100°C, cone gas flow at 50 L/h and desolvation gas flow at 800 L/h. Acquisition time was performed from m/z 100 to 2,000 Da.

### Enzyme-Linked Immunosorbent Assay (ELISA) and Immunohistochemistry on Tumor Sample

Xenograft tumors were harvested and embedded in paraffin blocks and cut into 4 μm thick tissue sections. The presence of tumor was confirmed on hematoxylin & eosin-stained slides (H&E). Proteins extracted from cryopreservation of liquid nitrogen of tumor tissues were used for ELISA experiment. The commercially available ELISA kits were used for protein expression detection of mouse IL-17 (Multisciences, Cat:EK2172), IL-2 (Multisciences, Cat:EK2022), IL-6 (Multisciences, Cat:EK2062), TGF-β (Multisciences, Cat:EK2812), IFN-γ(Multisciences, Cat:EK2802), and PD-1 (CUSABIO, Cat:CSB-E13586m). For immunohistochemistry staining assay, the paraffin embedded slides were dewaxed using xylene and rehydrated using alcohol of graded concentrations. Endogenous peroxidase activity was eliminated by 3% H_2_O_2_ for 15 min. The slides were then blocked with 5% goat serum for 20 min at 37°C, followed by IFN-γ primary antibody (Abcam, Cat: ab9657, 1:200 dilution) and IL-2 primary antibody (Abcam, Cat: ab180780, 1:125 dilution) incubation overnight at 4°C. Next day, each sample was then incubated with horseradish peroxidase-labeled secondary antibody for 1 h at room temperature, followed by staining using the ready-to-use reagent DAB kit. After dehydrating and drying, the sections were mounted with neutral gum and observed under a microscope. Images were acquired with a laser scanning microscope (LSM 700; Zeiss, New York, NY, United States), using 20× objective and processed with ZEN 2009 software (Zeiss, CA).

### CD4^+^ and CD8^+^ Were Detected by Flow Cytometry

The tissue sample preparation machine was homogenized, centrifuged, and the supernatant was stored. The PBMCs were labeled with two-color fluorescent labels according to manufacturer’s instruction. The number of CD4^+^ T cells and CD8^+^ T cells in each group was detected by flow cytometry.

### Statistical Analysis

Statistic calculations were performed using SPSS software (SPSS Inc., Chicago, IL, United States). Correlation between parameters was evaluated using the Pearson correlation coefficient. Student’s unpaired *t*-test was used to analyze the differences between the two groups, and the multiple test adjustments were performed using the Benjamini–Hochberg false discovery rate method. Each group was statistically significant with *P* < 0.05 as the smallest difference.

## Results

### Effect of PD-1 Antibody Immunotherapy on the Diversity of CT26 Tumor-Bearing Mice With Different Gut Microbiome

**FIGURE 1:**
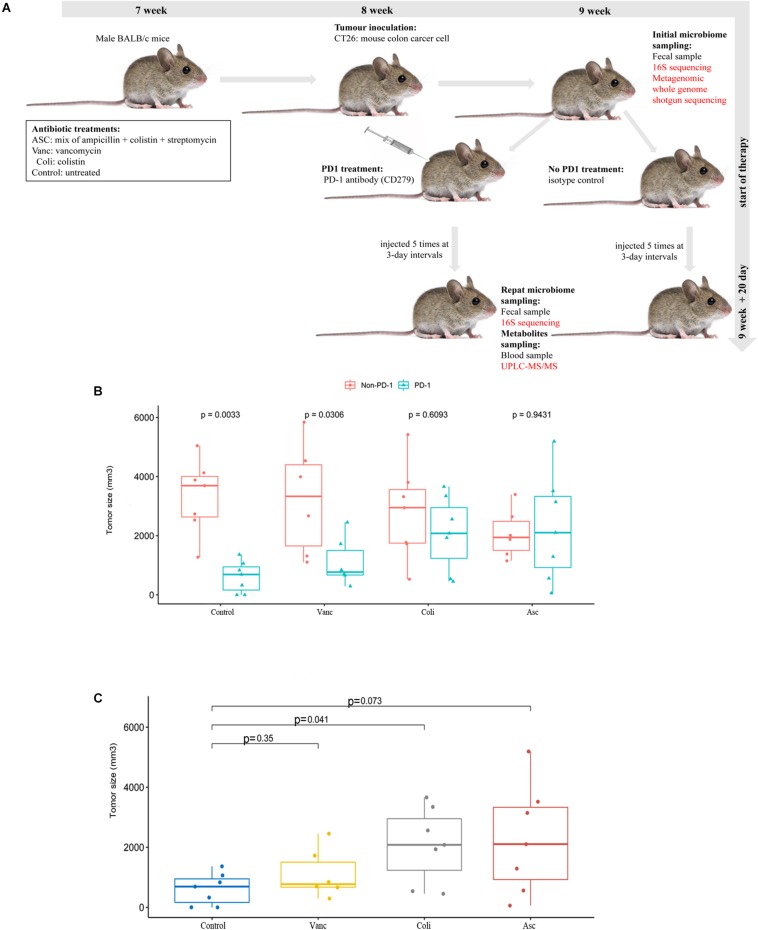
Effect of PD-1 antibody immunotherapy on the diversity of CT26 tumor-bearing mice with different gut microbiome. **(A)** Schematic diagram of mouse model experiment process and sample collection and analysis. **(B)** Tumor size of mice at the end of immunotherapy. **(C)** Tumor size of mice at the end of immunotherapy in the presence of different antibiotic regimen in each group.

Despite sharing the same genetic background, the CT26 tumor-bearing mice harbor different gut microbiota with different antibiotics, and the gut microbiome may play critical roles in the colon cancer development and anti-tumor immune responses. Therefore, we assessed the effects of the gut microbiome on PD-1 antibody immunotherapy by comparing tumor growth in CT26 tumor-bearing mice treated with different antibiotics (Figure 1A). We compared the relative therapeutic efficacy of PD-1 antibody against established CT26 tumor-bearing mice treated with vancomycin, colistin, ampicillin + streptomycin + colistin and non-antibiotic sterile drinking water. Tumor progression was controlled by anti-mouse PD-1 antibody compared with isotype control (Figure 1B). The result showed that the tumor growth was limited after injection of antibiotics when compared with the non-antibiotic sterile drinking water group. However, after adding PD-1 antibody treatment, the injection of antibiotics promoted tumor growth when compared with the non-antibiotic sterile drinking water group. Moreover, a combination of broad-spectrum antibiotics (ampicillin + streptomycin + colistin) compromised the antitumor effects of anti-mouse PD-1 (amp + strep + Coli group, non-response) (Figure 1C). Our results show that the anticancer effects of PD-1 antibody requires the gut microbiota. In particular, the anti-tumor effect of PD-1 antibody is well in the mice treated with non-antibiotic sterile drinking water (Control group, well-response), medium in the mice treated with vancomycin (Vanc group, medium-response), and poor in the mice treated with colistin (Coli group, poor-response).

### Gut Microbiome Diversity Is Associated With Response to PD-1 Antibody Immunotherapy in CT26 Tumor-Bearing Mice

**FIGURE 2:**
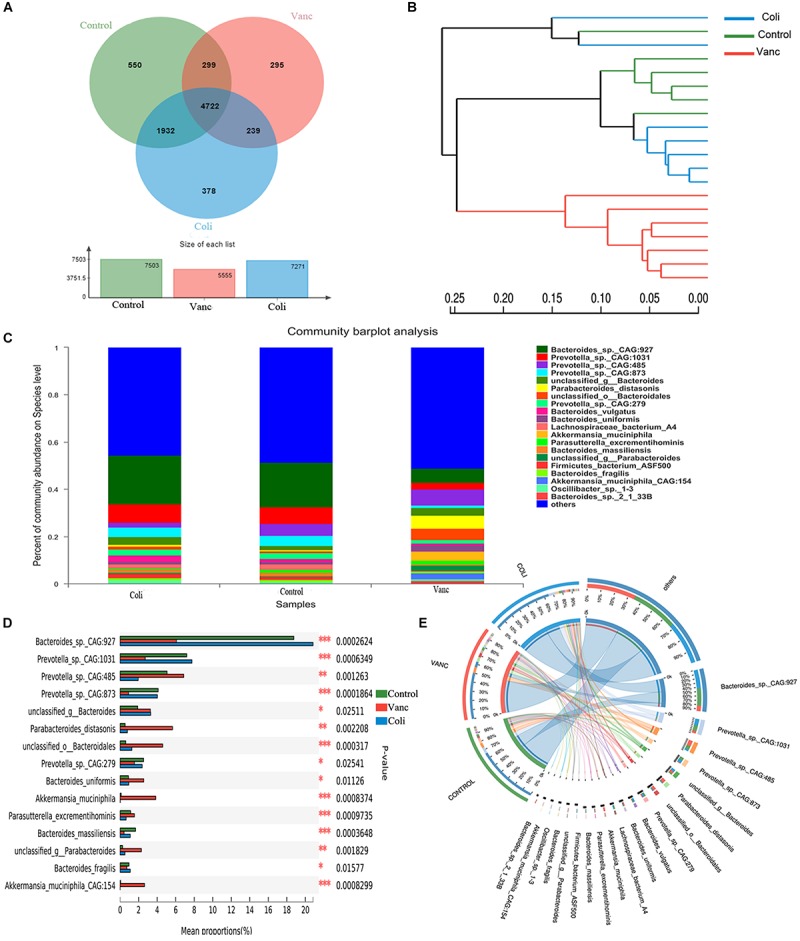
Gut microbiome diversity is associated with response to PD-1 antibody immunotherapy in CT26 tumor-bearing mice. **(A)** Venn diagram of the total number of species shared between the three groups. **(B)** Hierarchical clustering tree on OUT level. **(C)** The community map of dominant species in three group at the level of species, the columns with different colors represent different species, and the length of the columns represents the proportion of species. **(D)** Bar plot of compositional differences at species level in the gut microbiome of three groups of mice by one-way ANOVA. **(E)** Circos of sample and species: a visual circle diagram describing the correspondence between samples and species.

In order to better understand the compositional diversities in the microbiome can influence the response to immunotherapy, we prospectively collected gut (fecal) microbiome samples from CT26 tumor-bearing mice (*n* = 41) (Figure 1A and Supplementary Table S1). Fecal microbiome samples were collected at the initiation of PD-1 antibody immunotherapy and all available intestinal samples were classified by 16S rRNA gene sequencing, and metagenomic whole-genome shotgun (WGS) sequencing was performed on subset (*n* = 20). We first evaluated the gut microbiota landscape of all available samples (*n* = 20) by WGS sequencing, noting that the microbial communities of the three groups were relatively diverse. A total of 7,503 (Control group), 5,555 (Vanc group), and 7,271(Coli group) species were identified, and a total of 4,722 species were shared (Figure 2A). Hierarchical clustering analysis demonstrated a clear separation of community structure in Control group, Vanc group and Coli group, suggesting that these communities are distinct (Figure 2B). The community map reflected the abundance ratio and diversity of the species in each group at the species level, which could visually present the dominant species. As shown in the Figure 2C, *Bacteroides*_sp*._CAG:927* in the Control group (18.76%) and the Coli group (20.83%) accounted for the highest proportion of the population composition, followed by *Prevotella_*sp*.CAG: 1031* (Control group 7.19% vs. Coli group 7.75%). However, in the Vanc group, *Prevotella_*sp*._CAG:485* (6.87%) accounted for the highest proportion of the population composition, followed by *Bacteroides_*sp.*_CAG:927* (6.07%), Parabacteroides_distasonis (5.66%), unclassified_o__Bacteroidales (4.6%), Akkermansia_ muciniphila (3.85%), and Bacteroides_uniformis (2.55%). Compared with the Coli group, the population of the Vanc group is rich in species, and the proportion of each species is relatively average. Among the 16S sequencing results (Supplementary Figure S1), we demonstrated that distinct sets of bacterial taxa were associated with response to PD-1 antibody therapy, with enrichment of Bacteroidales_S24-7 in Control group, *Akkermansia_muciniphila* in Vanc group and *Bacteroides* in Coli group (Supplementary Figure S1). *Akkermansia_muciniphila* is the most abundant in the Vanc group, while the proportion of *Akkermansia_muciniphila* was 3.85% in the WGS sequencing analysis. The data after cluster analysis of species abundance indicated that on the Heatmap map, high-abundance and low-abundance species can be clustered. The similarity and difference of community composition of multiple samples at each classification level can be reflected by color gradient and similarity degree.

Based on the community abundance data obtained, the Wilcoxon rank-sum test was used to test the hypothesis of the species among the three groups (Control group, Vanc group Coli group) microbial communities, and to assess the significance level of the difference in species abundance and obtain the species with significant difference between groups (Figure 2D and Supplementary Figure S2). By performing multiple group comparison of the gut microbiome, with *Bacteroides_*sp.*_CAG:927* and *Prevotella_*sp.*_CAG: 1031* in the Control group and Coli group, *Prevotella_*sp._*CAG:485, Parabacteroides_distasonis, Akkermansia_muciniphila*, and *Bacteroides_uniformis* in the Vanc group. However, *Akkermansia_muciniphila* was significantly present in the Vanc group that compared with the other two groups (Figure 2D and Supplementary Figure S1B). The Circos analysis intuitively reflected the dominant species composition ratio of each sample at the species level, and also reflected the distribution ratio of each dominant species among different samples, and verified the community map analysis results (Figure 2E).

Our results indicate that in the context of different gut flora, with Bacteroides_sp._CAG:927 and Bacteroidales_S24-7 were enriched in Control group. Bacteroides_sp._CAG:927, Prevotella_sp._CAG: 1031, and Bacteroides were enriched in Coli group, Prevotella_sp._CAG:485 and Akkermansia_muciniphila were enriched in Vanc group. Therefore, it can be inferred that the diversity of microbial components may affect the effect of PD-1 antibody immunotherapy in the future, providing theoretical evidence for finding the status of gut microbiome suitable for PD-1 antibody immunotherapy.

### Metagenomic Analysis of Functional Diversity of Gut Microbiome

To investigate the underlying mechanism of the effects of the gut microbiome on PD-1 antibody therapeutic response and determine biologically significant differences, we determined changes in functional composition using the KEGG pathway database. Linear discriminant analysis of effect size (LEfSe) analysis was then also performed to explore KEGG pathways with significantly different abundances between Vanc group and Coli group.

**FIGURE 3:**
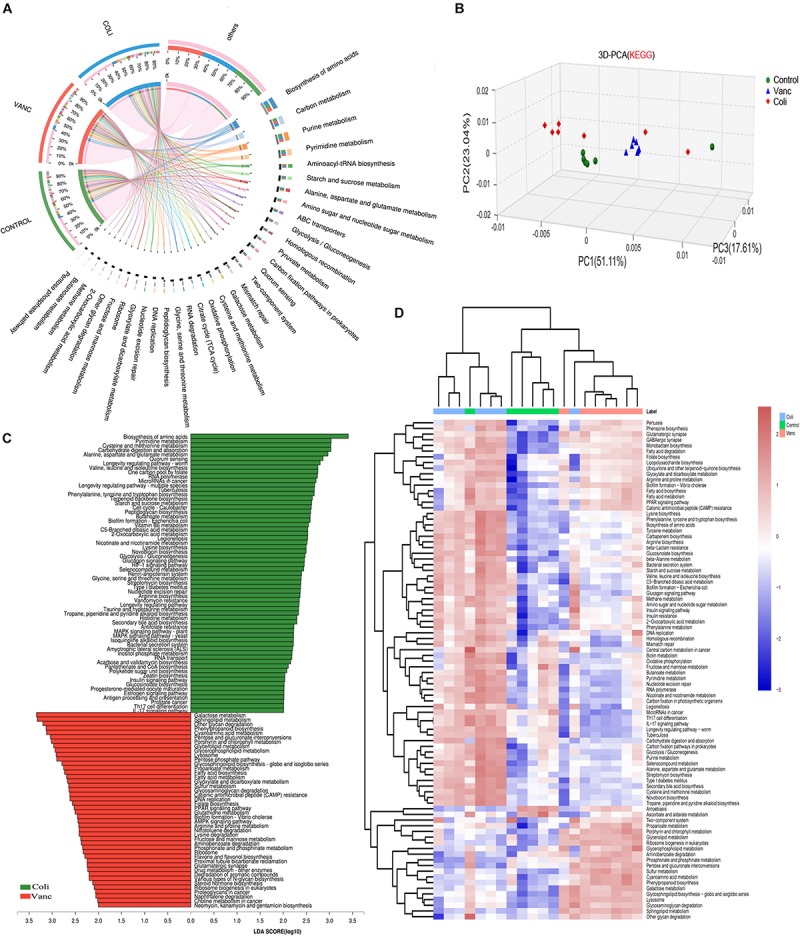
Metagenomic analysis of functional diversity of gut microbiota. **(A)** Circos of samples and functional KEGG pathways. **(B)** 3D-PCA for KEGG pathways. **(C)** LDA scores computed for differentially abundant taxa in the fecal microbiomes of Coli group and Vanc group. Length indicates effect size associated with a taxon. *p* = 0.05 for the Kruskal–Wallis test; LDA score > 3. **(D)** Unsupervised hierarchical clustering of KEGG pathways predicted in the metagenomes of fecal samples. Columns represent samples and rows represent enrichment of predicted KEGG pathways.

The result of 3D-PCA for KEGG pathways suggested that functional items seems to be clustering in Control group and Vanc group, which was consistent to the similar response to PD-1 antibody therapy in Control group and Vanc group (Figure 3B). The Circos analysis shows the abundance relationship between the sample and the function (Figure 3A). The result showed that used the threshold values (LDA > 2, *P* < 0.05), we found the multiple KEGG pathways were significantly enriched in Coli group, including multiple biosynthesis and metabolic of amino acids pathways, such as Pyrimidine metabolism, cysteine and methionine metabolism, alanine, aspartate, and glutamate metabolism, as well as immune-related pathways, such as Th17 cell differentiation and IL-17 signaling pathway, etc. (Figure 3C and Supplementary Figure S3). Meanwhile, also multiple KEGG pathways were significantly increased Vanc group (LDA > 2, *P* < 0.05), including galactose metabolism, sphingolipid metabolism, glyoxylate and dicarboxylate metabolism, cyanoamino acid metabolism, glycerolipid metabolism and PPAR signaling pathway, etc. (Supplementary Figure S4). Comparing the enrichment of pathways between the three groups, it was found that the metabolic function changed and unsupervised hierarchical clustering was performed. As shown in Figure 3D, synthetic and metabolic functions predominated in vanc-treated mice, including glycerolipid metabolism, sphingolipid metabolism, propanoate metabolism, galactose metabolism, glycosphingolipid biosynthesis-globo and isoglobo series, lipopolysaccharide biosynthesis. The results indicated that the several metabolic functional pathways (Type I diabetes mellitus and Antigen processing and presentation and Pathways in cancer) and immune-related pathways (Th17 cell differentiation and IL-17 signaling pathway) predominated in Coli group, which may resulting in abnormal T cell glucose metabolism (Granados et al., 2017) and chronic inflammation (Joerger et al., 2016) in the host mice and may account for the poor efficacy of immunotherapy. However, these functional pathways (glycerolipid metabolism, glycosphingolipid biosynthesis-globoand isoglobo series) predominated in Vanc group, which may be related to the better therapeutic effect of PD-1 antibody immunotherapy.

### Compositional Differences in the Gut Microbiome Are Relatively Stable Over Time

**FIGURE 4:**
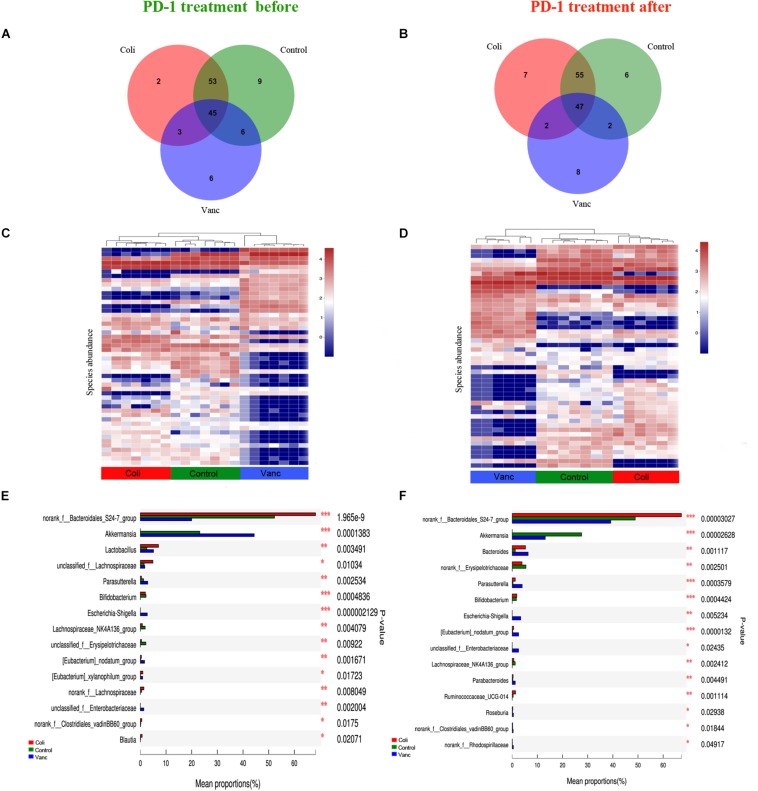
Compositional differences in the gut microbiome are relatively stable over time. **(A)** Venn diagram of the total number of species shared between the three subgroups in before PD-1 antibody treatment group. **(B)** Venn diagram of the total number of species shared between the three subgroups in after PD-1 antibody treatment group. **(C)** Unsupervised hierarchical clustering of top 50 species abundances in before PD-1 antibody treatment group. **(D)** Unsupervised hierarchical clustering of top 50 species abundances in after PD-1 antibody treatment group. **(E)** Bar plot of compositional differences at genus level in the gut microbiome of before PD-1 antibody treatment group mice by one-way ANOVA. **(F)** Bar plot of compositional differences at genus level in the gut microbiome of after PD-1 antibody treatment group mice by one-way ANOVA.

Next, we explored whether the bacterial composition and abundance in the gut microbiome are relatively stable over time in a limited longitudinal sample. We also collected gut microbiome samples from CT26 tumor-bearing mice (*n* = 40) at the end of PD-1 antibody immunotherapy (9 weeks + 20 days) and 16S rRNA gene sequencing was further performed (Figure 1A and Supplementary Table S1). The results indicated that there were 124 species and 127 species in before and after PD-1 antibody immunotherapy, respectively (Figures 4A,B). The species richness was not significantly different at the time before and after PD-1 antibody immunotherapy. Unsupervised hierarchical clustering was then performed with the species abundances. We found no significant changes in community distribution between the three groups (Control group, Vanc group, and Coli group) before and after PD-1 antibody immunotherapy (Figures 4C,D). To better understand compositional differences in the three distinct community types (Control group, Vanc group, and Coli group), we have again compared multiple groups of the gut microbiome and confirmed a community structure pattern that is very similar to that before and after PD-1 antibody immunotherapy, with *Bacteroidales_S24-7* in Control group and Coli group, Akkermansia_muciniphila in Control group and Vanc group (Figures 4E,F and Supplementary Figures S5, S6). Our results suggested that the bacterial composition and abundances in the gut microbiomes are comparatively stable over time in the limit longitudinal samples.

### Gut Microbiome Induces Specific Changes in Plasma Lipids and Metabolome

**FIGURE 5:**
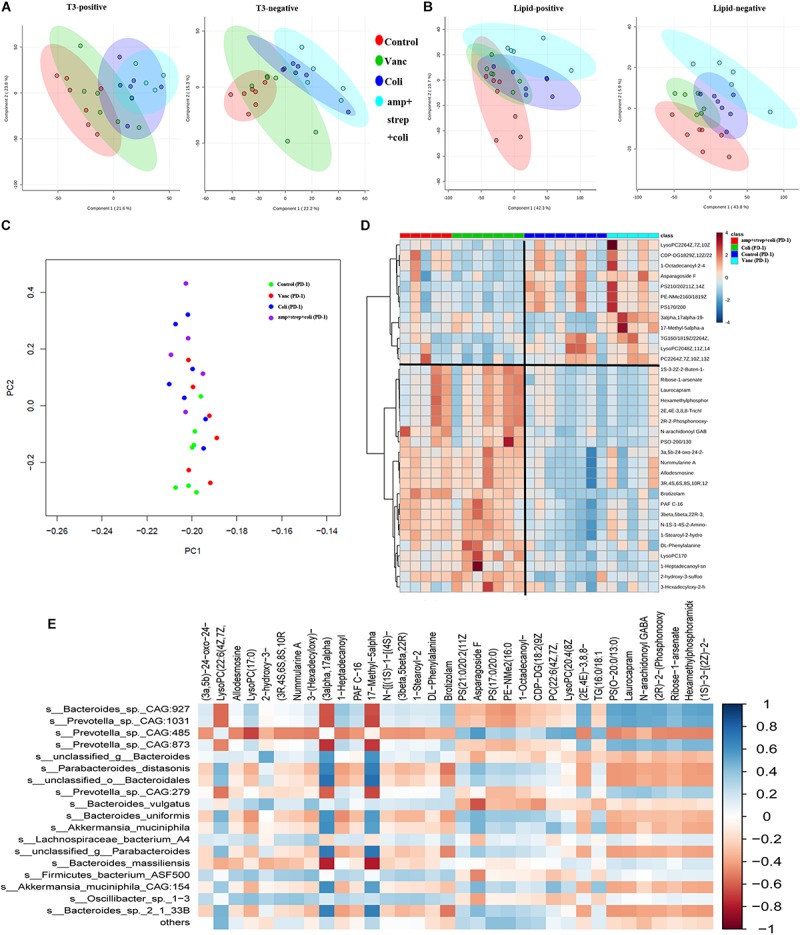
Gut microbiota induces specific changes in plasma lipids and metabolome. **(A)** PCA graphs of the plasma metabolome from QC samples in positive and negative metabolomics analysis. **(B)** PCA graphs of the plasma lipidome from all samples. **(C)** PCA of plasma metabolites from after PD-1 antibody treatment group mice. **(D)** Heatmap shows the normalized relative abundances of metabolites with annotation which were significantly changed in Control vs. Coli group and Vanc vs. Coli group. **(E)** Correlations between the gut microbiota (at the species level) and potential plasma compounds. Cells are colored based on Pearson correlation coefficient between predominant bacteria (relative abundance) and metabolites (normalized abundance) in plasma. The red color represents a significant negative correlation (*P* < 0.05), the blue color represents a significant positive correlation (*P* < 0.05).

Intestinal microbial products can induce physiological changes in the host without bacteria (e.g., weakening of colitis and anti-tumor effects). Therefore, we performed plasma metabolite profiling on the same mice (*n* = 48) by UPLC-MS to determine if we could detect significant differences in plasma metabolites. The PCA analysis was carried out based on the first two components. Positive and negative metabolomics analysis of positive and negative data collected PCA scores of QC samples closely clustered in the score map, showing good data quality and reproducibility of analytical methods (Figures 5A,B). Moreover, PCA analysis showed differences in the assignment of plasma metabolites in four group (Control group, Vanc group, Coli group, and Asc group) mice (Figures 5A,B).

In the PD-1 antibody treatment group mice, we further explore the metabolite perturbations in the four subgroups of mice (Control group, Vanc group, Coli group, and amp + strep + coli group). PCA analysis indicated that the plasma metabolites from control and Vanc group mice were clustered, whereas the plasma metabolites from coli and amp + strep + coli group mice were clustered (Figure 5C). Based on one-way ANOVA results, there were 214 metabolomics and 136 lipidomics significantly changed in control vs. Coli group and Vanc vs. Coli group. Accurate mass library searching identified 34 of these 350 metabolites (Supplementary Table S2). Further pathway enrichment showed that the 34 metabolites were involved in glycerophospholipid metabolism and Glycosylphosphatidylinositol (GPI)-anchor biosynthesis. Heatmap also indicated that the plasma metabolites of control and Vanc group were well separated from coli and amp + strep + coli group (Figure 5D).

To investigate the functional correlation between changes in gut microbiota and metabolite perturbations, a Pearson’s correlation matrix was generated by calculating the Pearson’s correlation coefficient between the taxa (at the species level) and candidate plasma compounds (Figure 5E). Significant correlation between disturbed gut microbiota and altered plasma metabolite profiles (*P* < 0.05). The correlation analysis showed that Prevotella_sp._CAG:485 was negatively correlated (*P* < 0.05, *r* < −0.5) with LysoPC (17:0), and they were down regulated in Control and Vanc group. LysoPC (20:4 (8Z,11Z,14Z,17Z)) was positively associated (*P* < 0.05, *r* > 0.5) with Prevotella_sp._CAG:485 and it was up-regulated in Control and Vanc group.

Bacteroides_sp._CAG:927 was positively correlated (*P* < 0.05, *r* > 0.5) with two metabolites and negatively correlated (*P* < 0.05, *r* < −0.5) with two metabolites. Of which, LysoPC (17:0) was positively associated with Bacteroides_sp._CAG:927, and it was down-regulated in Control and Vanc group (Figures 5D,E).

### Gut Microbiome Changes the Expression of Immune Factors in the Tumor Microenvironment

**FIGURE 6:**
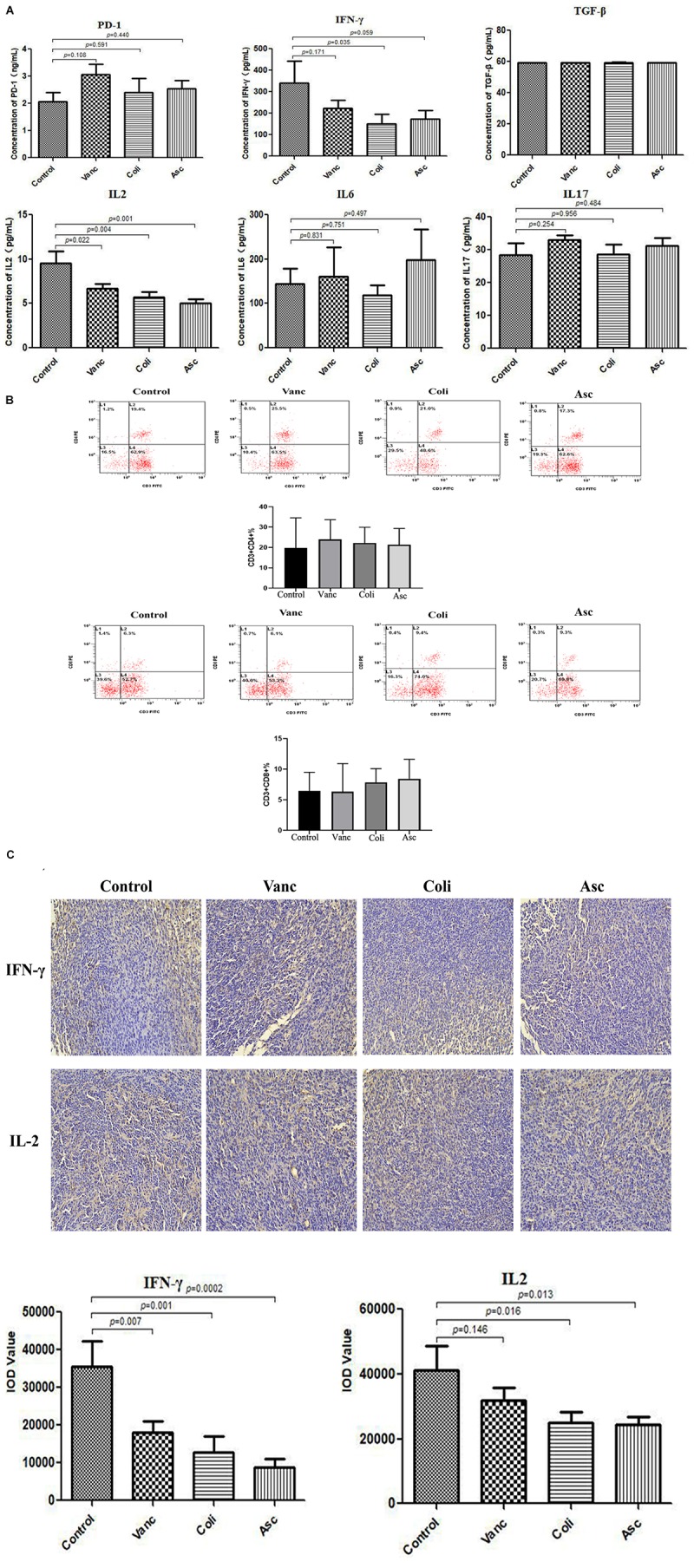
Gut microbiota changes the expression of immune factors in the tumor microenvironment. **(A)** Bar graph of PD-1, TGF-β, IFN-γ, IL-2, IL-6, and IL-17 in by Elisa tumor samples. **(B)** The number of CD4^+^ and CD8^+^ T cell were detected by flow cytometry. **(C)** Representative immunohistochemistry images on tumor samples.

PD-1 antibody immunotherapy response was closely related to changes in tumor microenvironment. We detected immune-related cytokines in tumor tissues by ELISA. The results revealed that was no difference in the expression of PD-1 protein in the tumor tissues of mice after PD-1 antibody treatment. In addition, TGF-β, IL6, and IL17 have no change significantly in the four groups (Control group, Vanc group, Coli group, and Asc group). IFN-γ was significantly differentially expressed in the coli-treated group (*P* = 0.035), and IL2 was significantly different in the Vanc group (*P* = 0.022), Coli group (*P* = 0.004), and Asc group (*P* = 0.001) (Figure 6A). We detected significant differences in cytokines (IFN-γ and IL-2) in four group by immunohistochemistry. We detected the number of CD4^+^ and CD8^+^ T cell by flow cytometry, the result showed the number of CD4^+^ and CD8^+^ T cell have no change significantly in the four groups (Control group, Vanc group, Coli group, and Asc group) (Figure 6B). From the results, the cell densities of IFN-γ and IL-2 were significantly lower in the other groups than in the Control group (Figure 6C), which was consistent with the ELISA results. Based on the above results, we speculated that the gut microbiome changes affect the expression of immune-related cytokines IFN-γ and IL-2 in the tumor microenvironment, resulting in a different therapeutic effect of PD-1 antibody.

## Discussion

Colorectal cancer is one of the common digestive tract tumors, and its morbidity and mortality are high and increasing year by year (Takiyama et al., 2017). Clinical outcomes have improved significantly through targeted therapies over the past few decades (Van Cutsem et al., 2016). In recent years, immunological CPIs have achieved significant results in the treatment of many solid tumors. However, only patients with dMMR/MSI can benefit partially from the treatment of CRC, this proportion of patients in all CRC patients is very low (Emambux et al., 2018). Therefore, how to make most patients with CRC benefit from immunotherapy is still a hot topic in this field. Studies have shown that during the immunotherapy of solid tumors, the different components of the host gut microbiome may affect the efficacy of immunotherapy at the level of tumor microenvironment (Sivan et al., 2015). In this study, we used the CRC model induced by MSS-type CT26 cells, which is classic type and has the characteristics of chromosomal instability. Our study is the first to present the gut microbial genome and plasma metabolomics characteristics of CT26 tumor-bearing mice with PD-1 antibody immunotherapy. Using a combination of 16S rRNA sequencing, metagenomic shotgun sequencing and non-targeted metabolomics, we were able to identify specific gut microbiota species and many plasma metabolites associated with PD-1 antibody immunotherapy in Control group (well-response), Vanc group (medium-response), Coli group (poor-response), and Asc group mice (non-response). Our results demonstrated that antibiotics treatment altered the taxonomic composition, functional metagenomics and metabolic profiles of the intestinal microbiome of CT26 tumor-bearing mice. Moreover, the results indicated that tumors in broad-spectrum antibiotics (Asc group mice) treated did not respond to PD-1 antibody immunotherapy, which suggested that having a normal gut microbiome is crucial for anti-tumor immune responses. Some data suggested that compositional differences of gut microbiome were closely associated with the response to PD-1 antibody immunotherapy.

At the species and genus level, Bacteroides_sp._CAG:927 and Bacteroidales_S24-7 were enriched in Control group. Bacteroides_sp._CAG:927, Prevotella_sp._CAG: 1031, and Bacteroides were enriched in Coli group, Prevotella_sp._CAG:485 and Akkermansia_muciniphila were enriched in Vanc group. Therefore, it may be inferred that the diversity of microbial components may affect the effect of PD-1 antibody immunotherapy, Prevotella_sp._CAG:485 and Akkermansia_muciniphila was related to the better therapeutic efficacy of immunotherapy and Bacteroides and Bacteroides_sp._CAG:927 was related to the poor therapeutic efficacy of immunotherapy. We also found that the enrichment of the *Akkermansia* belonging to the phylum Verrucomicrobia in the intestine is associated with better efficacy, which is consistent with a clinical study conducted by Routy et al. (2018) on immunotherapy for epithelial tumors. Recently, Akkermansia has been considered as a “beneficial bacteria” who responded positively to the immune CPI (Santoni et al., 2018). Experiments on mice showed that Akkermansia can strengthen the gut barrier function (Everard et al., 2013), normalize metabolic endotoxemia and adipose tissue metabolism (Lukovac et al., 2014), leading to improve the immune responses (Ottman et al., 2016). Akkermansia.muciniphila decreased serum ALT and AST and pro-inflammatory cytokines and chemokines (IL-2, IFN-γ, IL-12p40, MCP-1, MIP-1a, and MIP-1b) were substantially attenuated. The microbial population provides a new perspective on immune function in host diseases (Wu et al., 2017). However, *Bacteroides* belong to the major phylum Bacteroidetes, which is an “unfavorable bacterium” in our study and significantly increased in Coli group mice. PD-1 antibody immunotherapy has a negative response, resulting in a poor therapeutic effect. This is consistent with a clinical study by Gopalakrishnan et al. (2018) on the treatment of melanoma with PD-1 inhibitors. The results also showed that the abundance of Bacteroidales_S24-7_group was relatively high and significantly different in Control group, Vanc group and Coli group mice. Some members of Bacteroidales_S24-7_group have been shown to be IgA coated, suggesting that they may be targeted by the innate immune system (Palm et al., 2014; Bunker et al., 2015). Study on mice reported that Bacteroidales_S24-7_group is one major bacteria might release bacterial eDNA, which can decrease pro-inflammatory activity and exert immunomodulatory functions in the mouse small intestine (Qi et al., 2017).

More and more research evidences link the body’s immune system to the gut microbiome. The gut microbiota and its metabolites have been shown to affect immune function and immune homeostasis in the gut and system. Microorganism-derived short-chain fatty acids (SCFA) and biotransformed bile acids (BA) as ligand-specific cellular signaling receptors (such as GPRCs, TGR5, and FXR) or affect the immune system through epigenetic processes (Rizzetto et al., 2018). The gut microbiota produces many immunogenic substances. For example, complex lipopolysaccharides on the surface of Gram-negative bacteria cause an immune response in the stool (Macfarlane et al., 2009). The intestinal microbiota coordinates host immunity, helps maintain the intestinal environment balance, and suppresses inflammation (Shi et al., 2017). Studies have shown that gut microbiome plays an important role in the formation of the intestinal immune system. Therefore, we have used the method of metagenomics to further explore the mouse gut microbiome and predict the function, and have good consistency with the 16S rDNA sequencing results at the level of phylum and genus. The results show that Akkermansia may have a better therapeutic effect at the genus level, which echoes previous results that Akkermansia was involved in the regulation of inflammatory factors (Everard et al., 2013). However, no strains associated with poor therapeutic effects were found, which may be related to differences in the two sequencing methods. The results of the metagenomic pathway function prediction show that the tumor signaling pathway, antigen recognition and presentation, Th17 cell differentiation, IL-17 secretion, estrogen signaling pathway, pyrimidine metabolism, nucleic acid shearing and repair are related to the poor efficacy of immunotherapy; and the biosynthesis of steroid hormones, mannose, phenylpropane, beet red, carotene, and the like is related to better therapeutic effects. Among them, Th17 cell differentiation, IL-17 secretion, tumor signaling pathway, antigen recognition and presentation are all related to the immune status of the tumor microenvironment. Whether the gut microbiome regulates the tumor immune microenvironment through these pathways needs to be verified in subsequent studies.

Human gut microbiome contributes to human disease and health through the accumulation of microbial metabolites (Tremaroli and Bäckhed, 2012; Ray, 2017). In recent years, researches on gut microbial metabolomics have found that when the abundance of probiotics in the intestinal flora increases, the concentration of bacterial metabolites such as propionate and butyrate in the intestine increases, and these bacterial metabolites increase the activity of immune cells (Clavel et al., 2017; Everts, 2018). Our study used metabolomics to detect serum metabolites from mice in different treatment groups. Heatmap also indicated that the plasma metabolites of Control and Vanc group were well separated from Coli and Asc group. The results showed that the two metabolic pathways glycerophospholipid metabolism and Glycosylphosphatidylinositol (GPI)-anchor biosynthesis, which had a significant difference between the Coli and Asc group in the treatment with poor PD-1 inhibitors and the Control and Vanc group. Further analysis showed that the metabolite LysoPC (20:4 (8Z, 11Z, 14Z,17Z)) was associated with better therapeutic effects, whereas LysoPC (17:0), 1-Heptadecanoyl-sn-glycero-3-phosphocholine was associated with poor therapeutic effects, suggesting that phospholipid metabolism might be closely related to immunotherapy. Therefore, we speculated that changes in the intestinal flora cause changes in the metabolic state of the body. These metabolites may be used to predict the effects of PD-1 antibody immunotherapy, but the way in which the gut microbiome affects the metabolism of the body requires further experimental verification.

A large number of studies have confirmed that the human immune system has an important relationship with the gut microbiome, and affects the maturation of the innate immune system and the establishment of the acquired immune system (Johansson et al., 2015; Honda and Littman, 2016; Spiljar et al., 2017). However, in studies on immunotherapy, it was found that the composition of different gut microbiome affects the infiltration of immune cells in the tumor microenvironment, thereby regulating the immune state of the tumor microenvironment. This has been confirmed in a number of sterile animal experiments (Sivan et al., 2015; Vetizou et al., 2015; Matson et al., 2018). Our study showed that the expression of immune-related cytokines IFN-γ and IL-2 in the tumor microenvironment affects the therapeutic effect of PD-1. IFN-γ has antitumor and immunoregulatory effects. It regulates the expression levels of 30 genes and produces a variety of cellular responses (Sun et al., 2017). IFN-γ also promotes NK cell activity, antigen presentation and increases macrophage lysosomal activity (Zhou et al., 2013; Assani et al., 2014; Takeda et al., 2018). Studies have shown that the expression of IFN-γ was related to the intestinal microflora community (Fernández-Santoscoy et al., 2015). IL-2 can regulate the cellular activity of leukocytes in the immune system, react with antibodies, hematopoiesis and tumor surveillance, and induce killer cells to produce cytokines such as IFN-γ and TNF-α (Gui et al., 2016). IL-2 can also induce PBMC or tumor infiltrating lymphocytes (TIL) into lymphokine-activated killer cells *in vitro* (LAK) (Liu Z. et al., 2018). T-cell cytokine production (IL-2 and IFN-γ) and proliferation assay confirmed that a measurable PD1/PD-L1 signal was generated (Khedri et al., 2018). Blocking PD1/PD-L1 binding can significantly enhance the killing efficiency of effector-target cells, which may be related with promoting the release of IFN-γ and IL-2 (Liu Y.F. et al., 2018). Our previous study found that Gegen qinlian decoction enhances antitumor effects and PD-1 antibody efficacy by tuning intestinal microbiota in the mouse CT26 tumor model, the mechanism may be that Gegen qinlian decoction affects this process by regulating the glycerophospholipid metabolic pathway, accompanied by the expression levels of IFN-γ and IL-2 in the tumor were elevated (Lv et al., 2019). In our study, the better efficacy of PD-1 antibody in the treatment of MSS-type CRC was associated with the glycerophospholipid metabolic pathway, while the metabolomics metabolite pathway enrichment was enriched in the glycerophospholipid metabolic pathway, accompanied by intergroup changes in the expression levels of IFN-γ and IL-2 in the tumor microenvironment. According to our results, it is speculated that the gut microbiome changes affect the metabolism of glycerophospholipids in the body, thereby altering the expression of immune-related cytokines IFN-γ and IL-2 in the tumor microenvironment, resulting in a different therapeutic effect of PD-1 antibody.

Our results confirmed that the gut microbiome played a key role in the treatment of CT26 tumor-bearing mice with PD-1 inhibitors, especially the Prevotella_sp._CAG:485 and Akkermansia to PD-1 inhibitors have important significance in the treatment of CT26 tumor-bearing mice. In addition, the “favorable” gut microbiome (e.g., high abundance of *Akkermansia*) contributed to systemic and anti-tumor immune responses by enhancing anabolic functions, whereas the “unfavorable” gut microbiome (e.g., high abundance of *Bacteroides*) impaired immunity to self and tumor tissue mediated mainly by increased chronic inflammation, and it was speculated from the results that Bacteroidales_S24-7 and Bacteroides_sp._CAG:927 were essential for regulating the imbalance of gut microbiota. Metabolomics screened 34 known metabolites, and the metabolites were enriched in the glycerophospholipid metabolic pathway consistent with the metagenomic prediction pathway in Vanc group, it suggests that Prevotella_sp._CAG:485 and Akkermansia may maintain the normal efficacy of PD-1 antibody by affecting the metabolism of glycerolipid. However, due to the lack of validation of the target flora and the lack of confirmation of the exact relationship between the efficacy of the flora, serum metabolites and PD-1 inhibitors, a large number of studies are needed to further confirm the results of this experiment. Our findings highlight the therapeutic potential of modulating the gut microbiome in CRC mice receiving PD-1 antibody immunotherapy. Changes in gut microbiome lead to changes in metabolic level, which affect the expression of immune-related cytokines IFN-γ and IL-2 in the tumor microenvironment, resulting in a different therapeutic effect of PD-1 antibody.

## Conclusion

Collectively, our data showed that changes in the gut microbiome affect the glycerophospholipid metabolic pathway, thereby regulating the therapeutic potential of PD-1 antibody in the immunotherapy of MSS-type CRC tumor-bearing mice.

## Data Availability Statement

The original contributions presented in the study are publicly available. This data can be found here: https://www.ncbi.nlm.nih.gov/bioproject/ accession number PRJNA594254.

## Ethics Statement

The study was approved by Laboratory Animal Ethical Committee of Fourth Hospital Hebei Medical University (China), permission SCXK(JI)2013-0051. The animal use protocol has been reviewed and approved by the Laboratory Animal Ethical Committee Fourth Hospital Hebei Medical University.

## Author Contributions

ZL and XX conceived the investigations. JiL, YL, DJ, and LK planned the experiments. JinL conceived and led the bioinformatic analyses. YJ and CZ performed the cell culture and mice experiments. YZ and JiaL analyzed the antibiotic treatments experiments. FG and NW performed the sample collection. ZL and XX analyzed the data and wrote the manuscript.

## Conflict of Interest

The authors declare that the research was conducted in the absence of any commercial or financial relationships that could be construed as a potential conflict of interest.

## Abbreviations

CIM: CpG island methylation
CIN: chromosome instability
CMS: consensus molecular subtype
CPIs: checkpoint inhibitors
CRC: colorectal cancer
CRCSC: CRC Subtyping Consortium
DC: dendritic cells
dMMR: deficient in mismatch repair
eDNA: extracellular DNA
MDSCs: myeloid-derived suppressor cells
MSI: microsatellite instability
MSS: microsatellite stability
NK: natural killer
NR: non-redundant
PCA: principal coordinate analysis
PD-1: programmed death 1
pMMR: mismatch repair proficient
Treg: regulatory T cells
WGS: whole genome shotgun.
