# Supplementary material for: Gut Microbiome Influences the Efficacy of PD-1 Antibody Immunotherapy on MSS-Type Colorectal Cancer via Metabolic Pathway
Source: Front Microbiol. 2020 Apr 30;11:814. doi: 10.3389/fmicb.2020.00814 (PMC7212380; doi:10.3389/fmicb.2020.00814)

Supplementary Materials for

**Gut microbiome influences the efficacy of PD-1 antibody immunotherapy on MSS-type colorectal cancer via metabolic pathway**

Xinjian Xu^1^, Ji Lv^1,2^, Fang Guo^3^, Jing Li^4,5^, Yitao Jia^6^, Da Jiang^7^,Na Wang^3^,Chao Zhang^8^, Lingyu Kong^5^, Yabin Liu^1^, Yanni Zhang^1^, Jian Lv^1^, Zhongxin Li^1^*

^1^ Department of thoracic surgery, The Fourth Hospital of Hebei Medical University, 050035 Shijiazhuang, China.

^2^ Department of Surgery, The First Hospital of Qinhuangdao, 066000 Qinhuangdao, China

^3^ Department of Pharmacology, Hebei Medical University, 050017 Shijiazhuang, China

^4^Department of Traditional Chinese Medicine, The Fourth Hospital of Hebei Medical University, 050017 Shijiazhuang, China

^5^ College of Combine Traditional Chinese and Western Medicine, Hebei Medical University, 050017 Shijiazhuang, China

^6^ Third Department of Oncology, Hebei General Hospital, 050051 Shijiazhuang, China

^7^Department of Oncology, The Fourth Hospital of Hebei Medical University, 050035 Shijiazhuang, China.

^8^Department of Clinical Laboratory,The Fourth Hospital of Hebei Medical University, 050035 Shijiazhuang, China.

**Corresponding author**: Zhongxin Li. Second Department of surgery, The Fourth Hospital of Hebei Medical University, 169 Tianshan Road, Shijiazhuang, Hebei 050035, P.R. China. Email: Lizhongxin96@163.com Fax: +86-0311-66696390

**Supplementary Table 1 Availability of gut microbiome samples or blood samples by analyses**

|  | **Anti-mouse PD-1 treatment** | | | |  | **Non-PD-1 treatment** | | | |  |
| --- | --- | --- | --- | --- | --- | --- | --- | --- | --- | --- |
|  | Control | Vanc | Coli | Amp+Strep+Coli |  | Control | Vanc | Coli | Amp+Strep+Coli |  |
| Start of immunotherapy | n=7 | n=7 | n=7 | n=0 |  | n=6 | n=7 | n=7 | n=0 | 16S rRNA sequencing |
|  | n=6 | n=7 | n=7 | n=0 |  | n=0 | n=0 | n=0 | n=0 | Metagenomic shotgun sequencing |
| End of immunotherapy | n=7 | n=6 | n=6 | n=0 |  | n=7 | n=7 | n=7 | n=0 | 16S rRNA sequencing |
|  | n=7 | n=6 | n=6 | n=5 |  | n=7 | n=5 | n=7 | n=5 | Non-targeted metabolomics |

Control: non-antibiotic treatments; Vanc: vancomycin; Coli: colistin; Amp+Strep+Coli: ampicillin+streptomycin+colistin.

**Supplementary Table 2 Plasma lipids and metabolome differ significantly in Control vs. Coli group and Vanc vs. Coli group**

|  | **Compound ID** | **Compound name** | **p-value** |
| --- | --- | --- | --- |
| **T3-pos** | CSID118802 | (3alpha,17alpha)-19-Norpregn-5(10)-en-20-yne-3,17-diol | 1.39E-06 |
|  | 19420-57-6 | 1-Stearoyl-2-hydroxy-sn-glycero-3-phosphocholine | 4.03E-06 |
|  | 50930-23-9 | 1-Heptadecanoyl-sn-glycero-3-phosphocholine | 0.000794 |
|  | CSID391398 | N-{[(1S)-1-[(4S)-2-Amino-1,4,5,6-tetrahydro-4-pyrimidinyl]-2-{[(2S)-4-methyl-1-oxo-1-{[(2S)-1-oxo-3-phenyl-2-propanyl]amino}-2-pentanyl]amino}-2-oxoethyl]carbamoyl}-L-phenylalanine | 1.63E-06 |
|  | CSID2357 | Brotizolam | 3.92E-08 |
|  | 74389-68-7 | PAF C-16 | 0.000374 |
|  | CSID10252447 | (3beta,5beta,22R)-3,14,22,25-Tetrahydroxycholest-7-en-6-one | 3.50E-06 |
|  | CSID223205 | 17-Methyl-5alpha-androst-2-ene-1alpha,17beta-diol | 2.35E-08 |
|  | 19420-57-6 | 1-Stearoyl-2-hydroxy-sn-glycero-3-phosphocholine | 2.50E-06 |
|  | 150-30-1 | DL-Phenylalanine | 2.72E-08 |
| **T3-neg** | HMDB0002429 | (3a,5b)-24-oxo-24-[(2-sulfoethyl)amino]cholan-3-yl-b-D-Glucopyranosiduronic acid | 1.86E-05 |
|  | HMDB0010404 | LysoPC(22:6(4Z,7Z,10Z,13Z,16Z,19Z)) | 0.001173 |
|  | HMDB0127958 | 2-hydroxy-3-(sulfooxy)butanedioic acid | 0.014432 |
|  | HMDB0012108 | LysoPC(17:0) | 0.000688 |
|  | HMDB0040704 | Allodesmosine | 2.16E-07 |
|  | CSID4445390 | (3R,4S,6S,8S,10R,12R,14R,15R,16R,17E,19E,21E,23E,25E,27S,28R)-4,6,8,10,12,14,15,16,27-Nonahydroxy-3-[(1R)-1-hydroxyhexyl]-17,28-dimethyloxacyclooctacosa-17,19,21,23,25-pentaen-2-one | 1.63E-07 |
|  | HMDB0029336 | Nummularine A | 3.26E-07 |
|  | CSID3845 | 3-(Hexadecyloxy)-2-hydroxypropyl 2-(trimethylammonio)ethyl phosphate | 2.86E-06 |
| **Lipid-pos** | CSID4444461 | (2E,4E)-3,8,8-Trichloro-7-(4-chlorophenyl)-2-hydroxy-6-oxo-2,4,7-octatrienoic acid | 0.03568 |
|  | HMDB0010396 | LysoPC(20:4(8Z,11Z,14Z,17Z)) | 0.003068 |
|  | LMGP01050056 | PC(22:6(4Z,7Z,10Z,13Z,16Z,19Z)/0:0) | 0.005206 |
|  | LMGL03010925 | TG(16:0/18:1(9Z)/22:6(4Z,7Z,10Z,13Z,16Z,19Z))[iso6] | 5.61E-07 |
|  | CSID391511 | (2R)-2-(Phosphonooxy)-3-sulfopropanoic acid | 0.037739 |
|  | HMDB0012285 | Ribose-1-arsenate | 0.031566 |
|  | CSID39176 | Laurocapram | 0.053149 |
|  | LMFA08020074 | N-arachidonoyl GABA | 5.69E-05 |
|  | CSID4444880 | (1S)-3-[(2Z)-2-Buten-1-yl]-2-methyl-4-oxo-2-cyclopenten-1-yl (1R,3R)-3-[(1E)-3-methoxy-2-methyl-3-oxo-1-propen-1-yl]-2,2-dimethylcyclopropanecarboxylate | 0.039546 |
|  | CSID12158 | Hexamethylphosphoramide | 0.048386 |
|  | LMGP03020042 | PS(O-20:0/13:0) | 0.022574 |
| **Lipid-neg** | 123063359 | PS(21:0/20:2(11Z,14Z)) | 0.013387 |
|  | LMGP02010322 | PE-NMe2(16:0/18:1(9Z)) | 0.001477 |
|  | 123063628 | PS(17:0/20:0) | 0.000799 |
|  | HMDB0029321 | Asparagoside F | 0.018203 |
|  | HMDB0115976 | CDP-DG(18:2(9Z,12Z)/22:6(4Z,7Z,10Z,13Z,16Z,19Z)) | 0.000388 |
|  | 96998-01-5 | 1-Octadecanoyl-2-(4Z,7Z,10Z,13Z,16Z,19Z-docosahexaenoyl)-sn-glycero-3-phosphoethanolamine | 0.000159 |

**Supplementary Figure 1.Compositional differences in the gut microbiome are associated with responses to PD-1 antibody immunotherapy** (A) Stacked bar plot of phylogenetic composition of common bacterial taxa at the species level in fecal samples at immunotherapy initiation. (B) Bar plot of compositional differences at genus level in the gut microbiome of three groups of mice by one-way ANOVA.

**
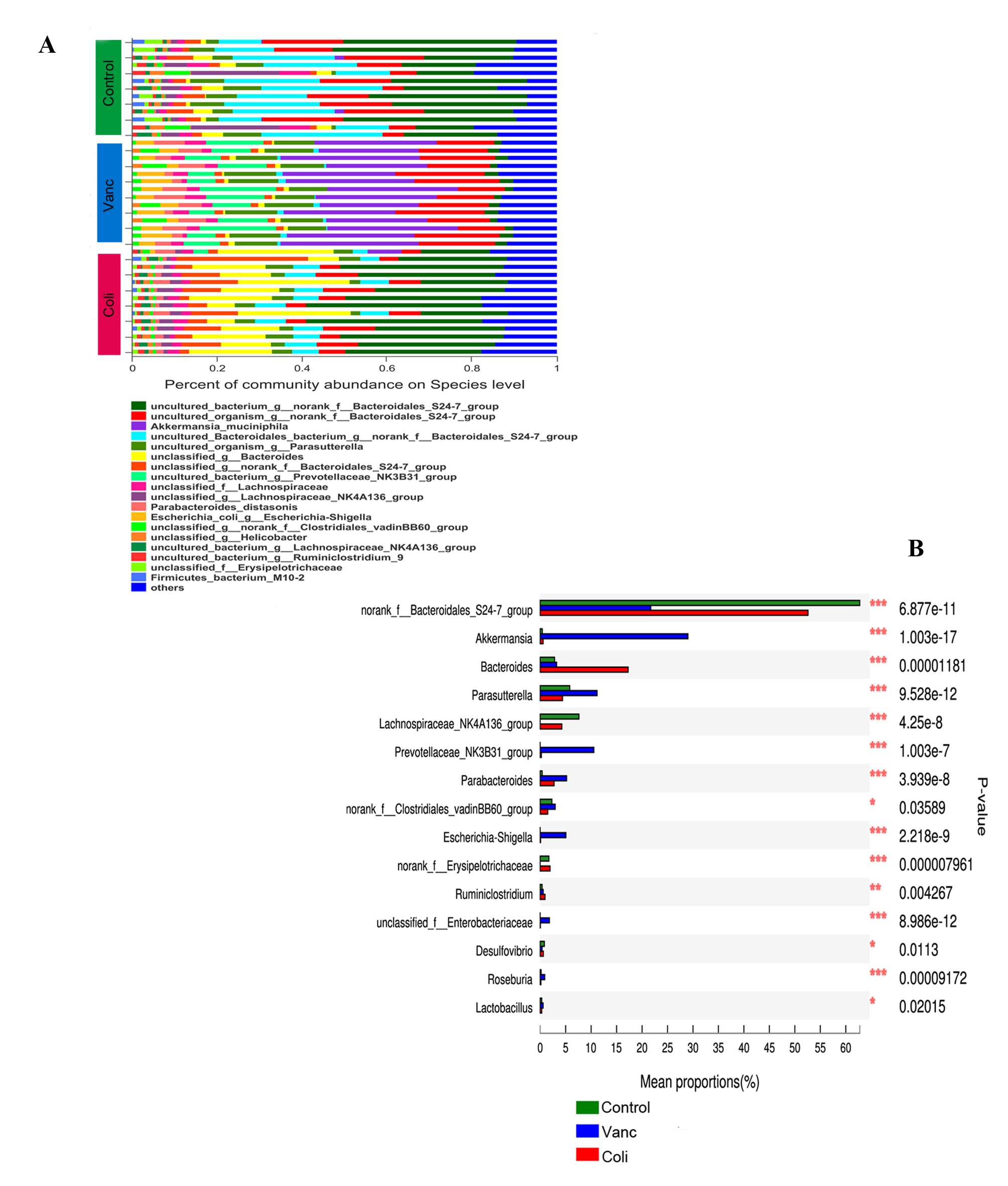
**

**Supplementary Figure 2.** Bar plot of compositional differences at species level in the Control vs. Coli,

Control vs. Vanc and Vanc vs.Coli groups of mice before PD-1 antibody treatment by one-way ANOVA.

**
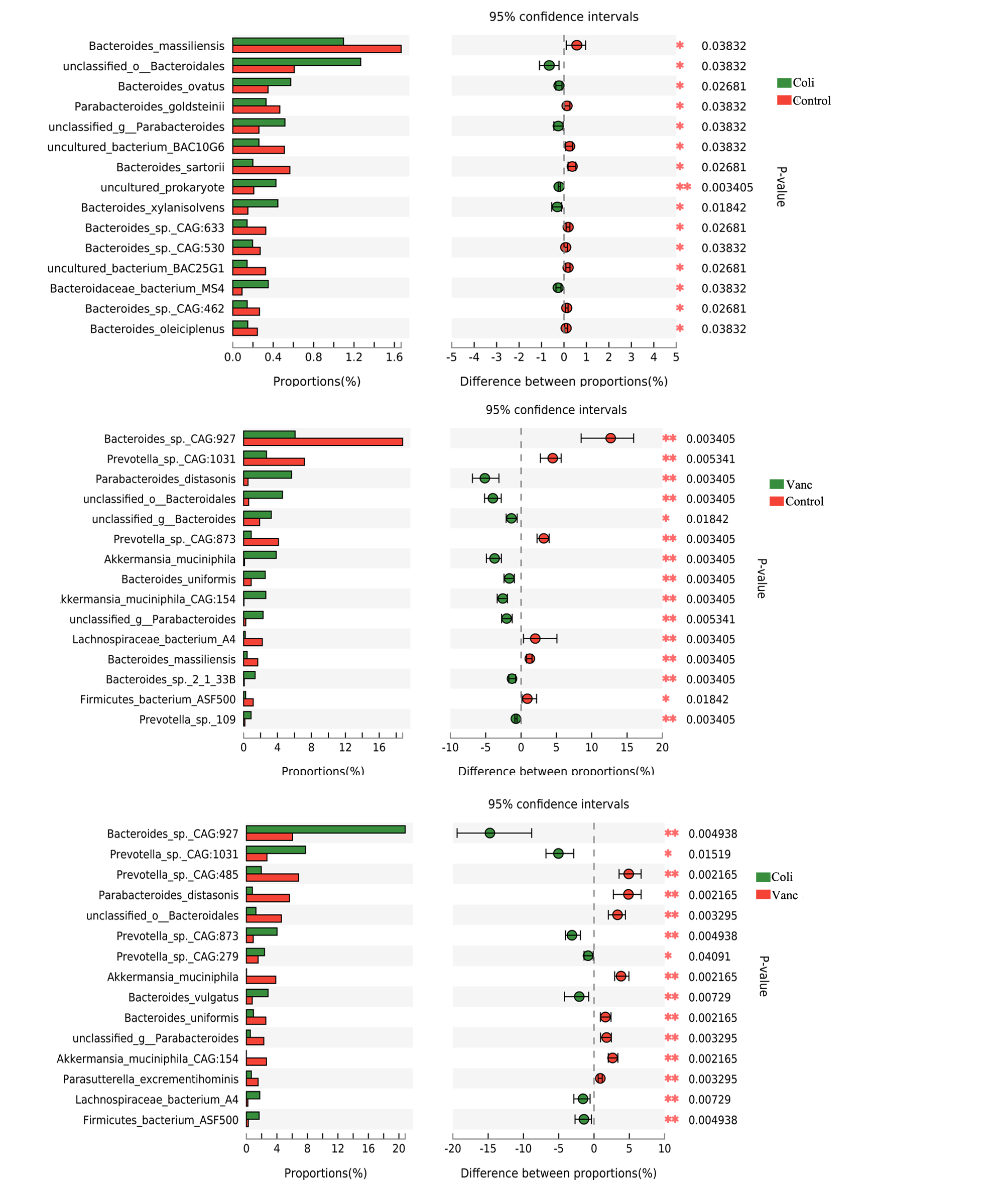
**

**Supplementary Figure 3**. LDA scores computed for differentially-abundant taxa in the fecal microbiomes of Control group and Coli group. Length indicates effect size associated with a taxon. p=0.05 for the Kruskal-Wallis test; LDA score > 3.


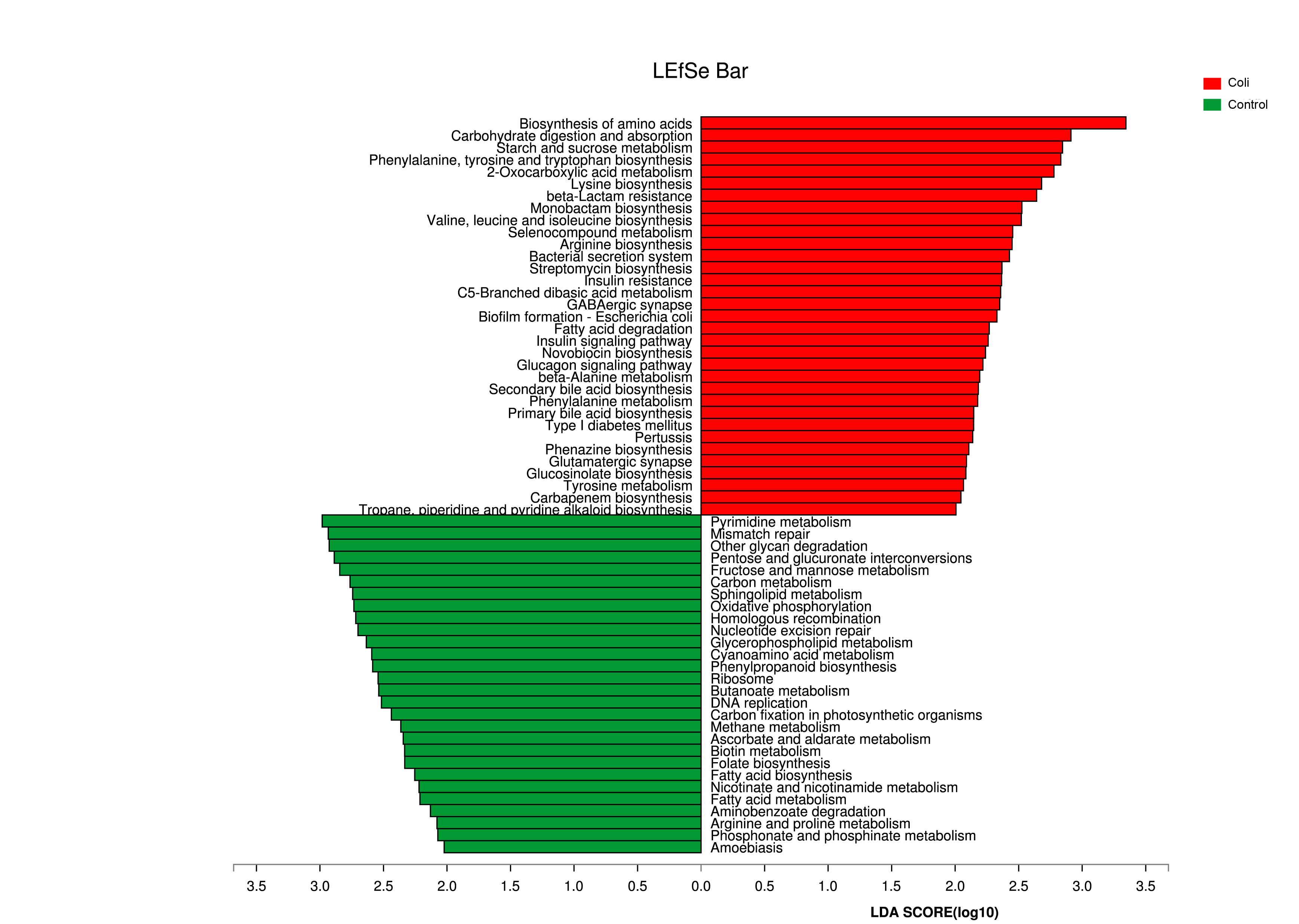


## **Supplementary Figure 4.** LDA scores computed for differentially-abundant taxa in the fecal microbiomes of Control group and Vanc group. Length indicates effect size associated with a taxon. p=0.05 for the Kruskal-Wallis test; LDA score > 3.


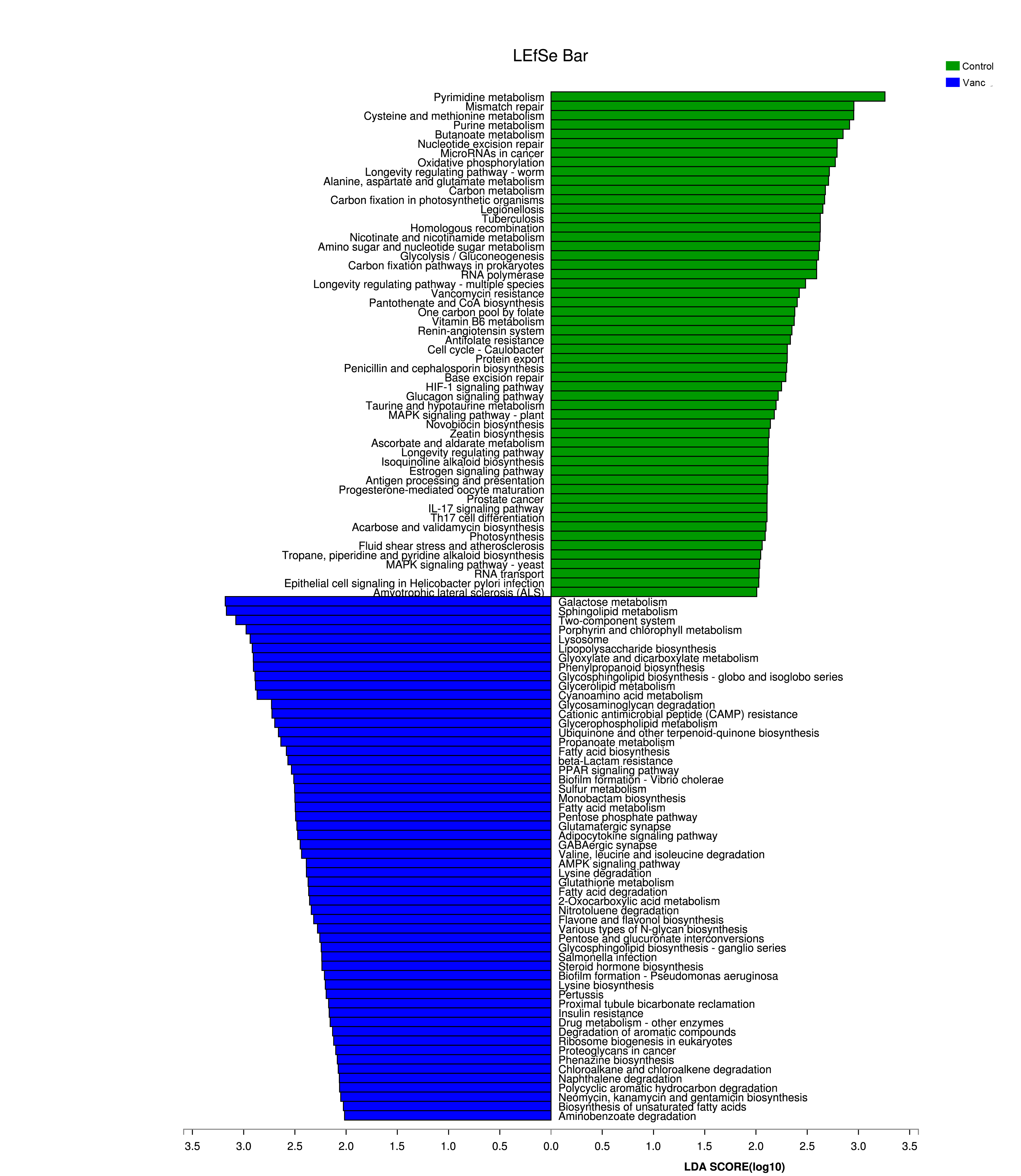


**Supplementary Figure 5**. Bar plot of compositional differences at genus level in the gut microbiome of Control vs. Coli,Control vs. Vanc and Vanc vs.Coli before PD-1 antibody immunotherapy by Student’s t test (equal variance). P-value<0.05. Only the top 15 microbiota were displayed.


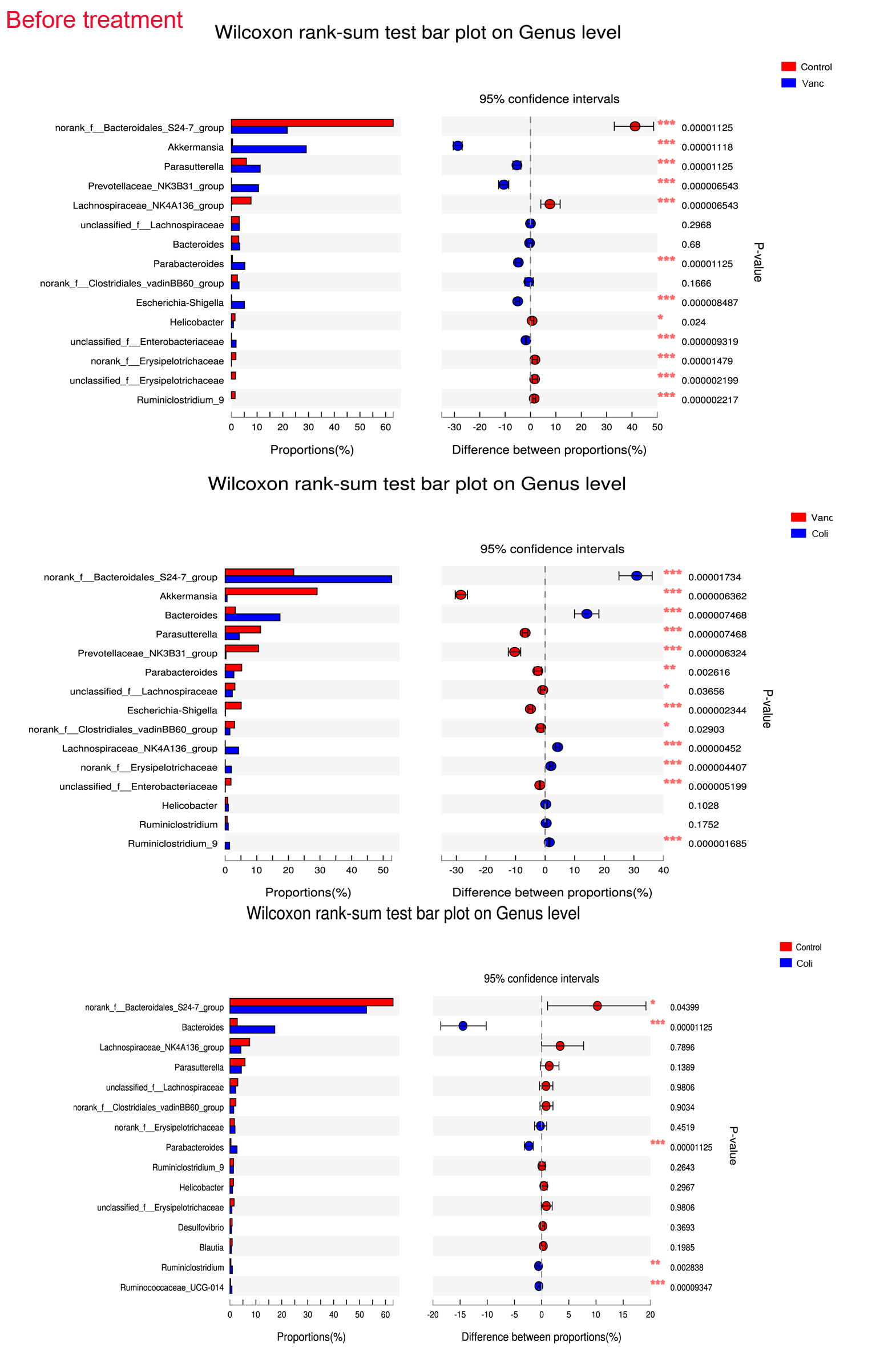


**Supplementary Figure 6**. Bar plot of compositional differences at genus level in the gut microbiome of Control vs. Coli,Control vs. Vanc and Vanc vs.Coli after PD-1 antibody immunotherapy (PD-1 treatment) by Student’s t test (equal variance). P-value<0.05. Only the top 15 microbiota were displayed.


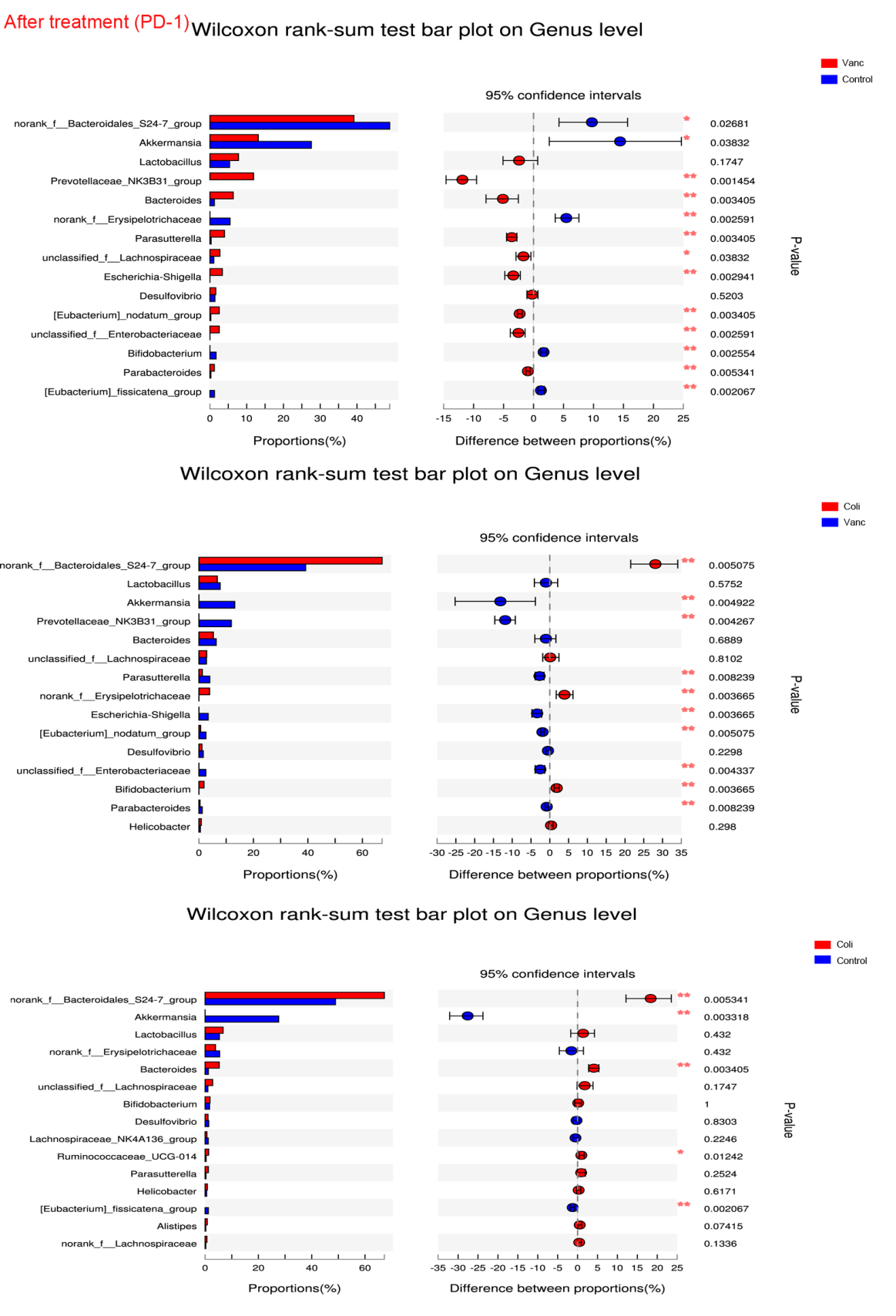

Supplement: Supplementary file 1 [file Data_Sheet_1.docx]
